# Cardioprotective and Antifibrotic Effects of Low-Dose Renin–Angiotensin–Aldosterone System Inhibitors in Type 1 Diabetic Rat Model

**DOI:** 10.3390/ijms242317043

**Published:** 2023-12-01

**Authors:** Dora B. Balogh, Agnes Molnar, Arianna Degi, Akos Toth, Lilla Lenart, Adar Saeed, Adrienn Barczi, Attila J. Szabo, Laszlo J. Wagner, Gyorgy Reusz, Andrea Fekete

**Affiliations:** 1 MTA-SE Lendület “Momentum” Diabetes Research Group, 1083 Budapest, Hungary; dorabiankabalogh@gmail.com (D.B.B.); akostoth95@gmail.com (A.T.); lenart.lillaa@gmail.com (L.L.); adaramedi992@gmail.com (A.S.); 2 Pediatric Center, MTA Center of Excellence, Semmelweis University, 1083 Budapest, Hungary; magnes0204@gmail.com (A.M.); degiarianna@gmail.com (A.D.); szabo.attila@semmelweis.hu (A.J.S.); reuszgs@gmail.com (G.R.); 3 Medical Imaging Center, Semmelweis University, 1082 Budapest, Hungary; adriennbarczi@gmail.com; 4 Department of Surgery, Transplantation and Gastroenterology, Semmelweis University, 1082 Budapest, Hungary; laszlo.drwagner@gmail.com

**Keywords:** diabetes, diabetic cardiomyopathy, heart failure, renin–angiotensin–aldosterone system inhibitors, myocardial fibrosis, intima–media thickness, pulse wave velocity

## Abstract

Diabetic cardiovascular complications are associated with up to 50% mortality, and current therapies are not effective enough. Renin–angiotensin–aldosterone system inhibitors (RAASis) are the standard of care for diabetic patients with hypertension and albuminuria. Based on our previous studies reporting the renoprotective effects of low-dose RAASis, here, we hypothesized that low-dose RAASi treatment has cardioprotective and antifibrotic benefits in type 1 diabetes mellitus (T1DM). After five weeks of T1DM, adult male Wistar rats received low doses of ramipril, losartan, or eplerenone for two weeks. Heart rate, blood pressure, and pulse wave velocity (PWV) were recorded. Aortic intima–media thickness (IMT), collagen accumulation, and myocardial fibrosis were assessed. All RAASis reduced PWV elevation, prevented the progression of myocardial fibrosis, and normalized B-type natriuretic peptide, troponin I, and fibroblast growth factor 23 levels without affecting blood pressure. Interestingly, only eplerenone reversed the decline in Klotho levels and reduced IMT and fibrosis in the media of the aorta. Our comparative analysis suggests that mineralocorticoid receptor antagonists, particularly eplerenone, may offer superior efficacy in halting both the arterial and the myocardial injuries in T1DM compared to angiotensin-converting enzyme inhibitors or angiotensin II type 1 receptor blockers.

## 1. Introduction

Diabetic patients have an increased risk of both atherosclerotic cardiovascular (CV) complications and diabetic cardiomyopathy (DCM), leading to heart failure (HF), which is associated with up to 50% mortality [1]. Moreover, about 40% of diabetic patients develop chronic kidney disease (CKD), which further increases CV mortality. Type 1 (T1DM) and type 2 diabetes mellitus (T2DM) have similar impacts; however, T1DM patients lose even more life years due to CV events [2,3].

Development of DCM is independent of hypertension or coronary heart disease. The underlying pathological factors are complex and include metabolic disturbances, systemic and cardiac inflammation, oxidative stress, hypoxia, and the overactivation of the renin–angiotensin–aldosterone system (RAAS) [4,5]. The increased angiotensin II (AngII) and aldosterone activity promotes cardiomyocyte loss, cell hypertrophy, inflammation, and extensive myocardial fibrosis, leading to cardiac remodeling and atherosclerosis [6,7].

Accelerated arterial stiffening due to prolonged hyperglycemia and metabolic changes further contributes to the elevated risk of CV diseases in diabetic patients. Carotid–femoral pulse wave velocity (PWV) is the gold standard non-invasive method for measuring central arterial stiffness, with a 1 m/s increment corresponding to a 15% increase in the risk of vascular events and mortality [8]. Intima–media thickness (IMT) is another crucial, non-invasive surrogate marker for vascular damage and atherosclerosis. It is recommended as a routine examination for identifying macrovascular risk in diabetic patients [9].

Pre-existing cardiovascular disease, especially chronic HF, can also increase the risk of CKD (type 2 cardiorenal syndrome). This is a bidirectional relationship, where the presence of one condition can provoke or exacerbate the other; CKD may lead to HF as well (type 4 cardiorenal syndrome) [10]. Both HF and CKD can be caused and aggravated by the presence of DM, underlining the close connection of cardiac–renal–metabolic diseases.

Current treatment strategies are not effective enough to prevent the progression of CV disease in DM. Though sodium–glucose cotransporter-2 (SGLT2) inhibitors are beneficial for preventing HF in T2DM, they are not suitable for treating T1DM patients because of the increased risk of ketoacidosis. RAAS is the other major pharmacological target in cardiovascular medicine. RAASis are the gold standard therapy for all diabetic patients; however, they are used exclusively when hypertension and albuminuria are present [11] or for diabetic patients with macrovascular complications plus hypertension, HF, or CKD [12,13]. RAASis are not recommended for diabetic normotensive patients without pre-existing CV complications, and clinical investigations with low-dose RAASis in T1DM-related CV complications are lacking.

Experimental data in the literature are also limited; therefore, to address this gap, we designed a study to explore the potential benefits of RAASis in T1DM-related CV diseases. We hypothesized that low-dose RAASis could improve myocardial damage, cardiac fibrosis, and vascular function in the streptozotocin-induced rodent model of DCM.

## 2. Results

### 2.1. Mean Arterial Pressure, Heart Rate, and Heart-to-Body Weight Ratio Were Unaffected by RAASi Treatment

Systolic and diastolic pressures and heart rate were measured, and the mean arterial pressure (MAP) was calculated. After seven weeks of T1DM, MAP remained unaltered in all groups. In line with the literature and our previous studies, STZ-induced diabetes was associated with a reduction in heart rate and increased heart-to-body weight ratio [14,15,16,17]. These parameters were unaffected by RAASis (Table 1).

### 2.2. RAASis Mitigated Diabetic Macrovascular Impairment

**Figure 1:**
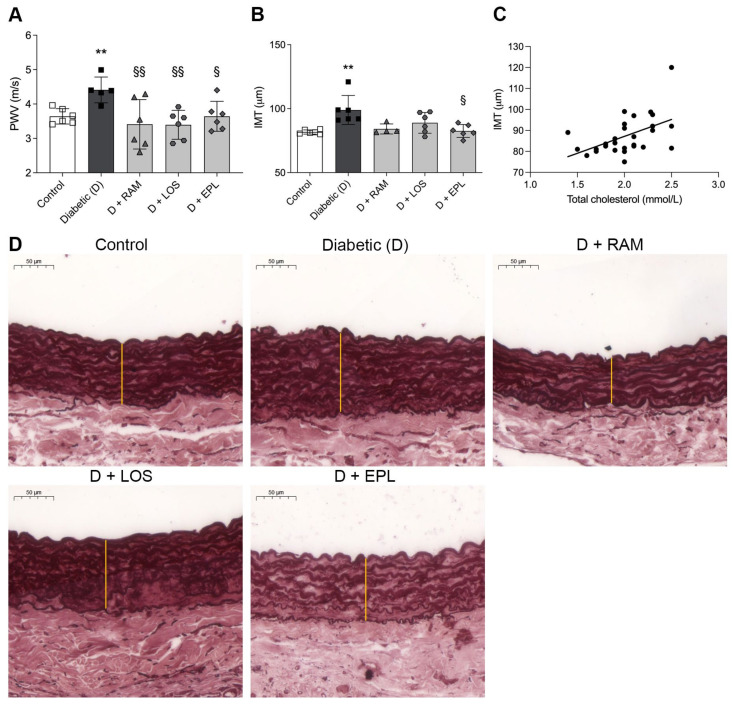
Pulse wave velocity (PWV) and aortic intima–media thickening (IMT) of control, diabetic (D), ramipril (D + RAM)-treated, losartan (D + LOS)-treated, or eplerenone (D + EPL)-treated diabetic rats: (**A**) evaluation of PWV; (**B**) the quantitative evaluation of IMT; (**C**) aortic IMT strongly correlates with serum total cholesterol level (R^2^ = 0.2532, *p* = 0.0064); (**D**) representative Orcein-stained aorta sections. Elastic fibers are stained brown after Orcein staining. They are visualized as either thin fibers or elastic lamella. Original magnification, ×40. Scale bar: 50 μm. Bars indicate means ± SDs. ** *p* < 0.01 vs. control, ^§^ *p* < 0.05 vs. diabetic, ^§§^ *p* < 0.001 vs. diabetic (*n* = 6/group).

Arterial stiffening is one of the earliest detectable manifestations of pathophysiological vascular remodeling. We observed elevated PWV values in diabetic rats, indicative of increased arterial stiffness (Figure 1A). The administration of each RAASi effectively reversed this elevation, restoring the PWV values to control levels.

Aortic IMT is an important early indicator of atherosclerosis, which changes progressively with disease duration, indicating its potential as an evolving biomarker [18]. The histological examination of aortic IMT revealed distinct characteristics in the control and diabetic rats. The control rats showed a prominent wavy internal elastic lamina, while the diabetic rats exhibited intimal thickening, irregularities, and diffused elastic membranes. RAASis lowered IMT increment and pathological changes in the aorta of diabetic rats, but only eplerenone reached the level of significance (Figure 1B,D). IMT significantly correlated with serum total cholesterol values (R^2^ = 0.2532, *p* = 0.0064) (Figure 1C).

### 2.3. RAASis Normalized the Levels of Specific Biomarkers of Myocardial Injury

**Figure 2:**
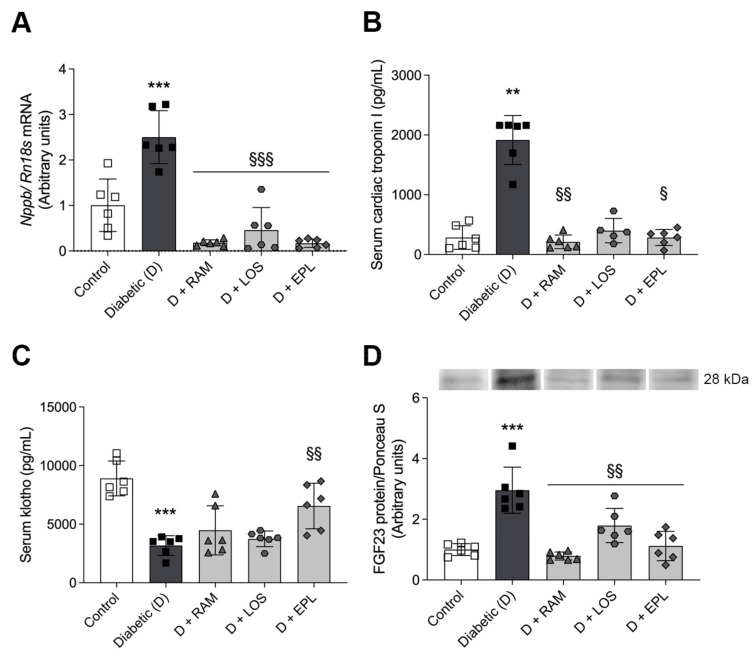
Specific biomarkers of myocardial injury of control, diabetic (D), ramipril (D + RAM)-treated, losartan (D + LOS)-treated, or eplerenone (D + EPL)-treated diabetic rats: (**A**) mRNA expressions of B-type natriuretic peptide (*Nppb*). mRNA expressions were normalized to Rn18S mRNA expression; (**B**) the quantitative evaluation of serum cardiac troponin I level; (**C**) the quantitative evaluation of serum Klotho levels; (**D**) protein levels of fibroblast growth factor 23 (FGF23). Proteins were normalized to total protein using Ponceau S staining as loading control. Bars indicate means ± SDs. ** *p* < 0.01 vs. control, *** *p* < 0.001 vs. control, ^§§^ *p* < 0.01 vs. diabetic, ^§§§^ *p* < 0.001 vs. diabetic (*n* = 6/group), ^§^ *p* < 0.05.

The mRNA expressions of BNP (*Nppb*)—specific to chronic left ventricular (LV) expansion—increased in the LV of diabetic rats (Figure 2A). In parallel, troponin I, a molecular marker of myocardial injury, was also elevated in the serum of diabetic rats. RAASi treatment decreased both to control levels (Figure 2B).

We observed decreased serum levels of Klotho in diabetic rats. This decline was reversed only by eplerenone treatment. LV protein levels of fibroblast growth factor 23 (FGF23) were elevated in the diabetic animals, and all RAASis minimized its levels (Figure 2C,D, respectively).

### 2.4. Eplerenone Decreased Fibrosis in the Media of the Aorta in Diabetic Rats

**Figure 3:**
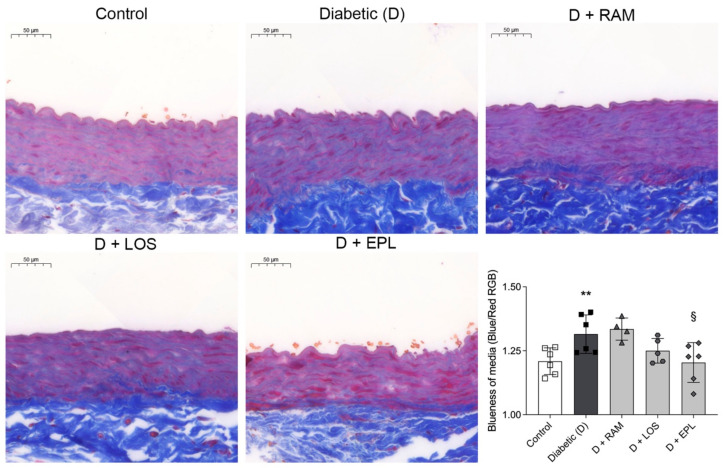
Representative Masson’s trichrome stained aorta sections and quantitative evaluation of fibrosis of control, diabetic (D), ramipril (D + RAM)-treated, losartan (D + LOS)-treated, or eplerenone (D + EPL)-treated diabetic rats. Original magnification, ×40. Scale bar: 50 μm. Bars indicate means ± SDs. ** *p* < 0.01 vs. control, ^§^ *p* < 0.05 vs. diabetic (*n* = 6/group).

Masson’s trichrome staining was performed to evaluate the fibrotic tissue accumulation in the media of the aortas. The control rats showed typical aortic wall structure, while the diabetic rats exhibited a disorganized elastic lamina arrangement. An extensive accumulation of extracellular matrix (ECM) components (blue staining) was observed in the aortas of diabetic rats, and only eplerenone significantly reduced the amount of fibrotic tissue (Figure 3).

### 2.5. RAASis Halted the Progression of T1DM-Induced Myocardial Fibrosis

**Figure 4:**
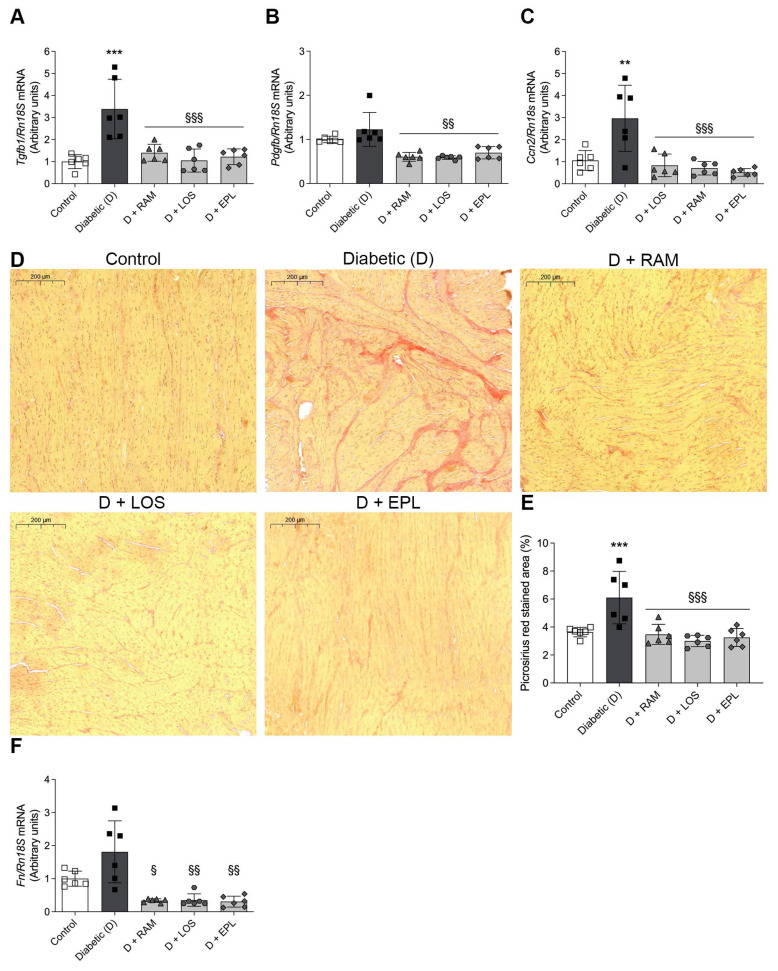
RAASi attenuate fibrosis in the left ventricle of diabetic rats. (**A**–**C**) mRNA expression of transforming growth factor β (*Tgfb1*), platelet-derived growth factor (*Pdgfb*), and connective tissue growth factor (*Ccn2*) of control, diabetic (**D**), ramipril (D + RAM)-treated, losartan (D + LOS)-treated, or eplerenone (D + EPL)-treated diabetic rats. (**D**,**E**) Representative picrosirius red stained left ventricular sections and quantitative evaluation of collagen accumulation. The red-stained area was measured based on the red color intensity. The myocardial field of measurement was manually selected taking caution to avoid the endocardium and perivascular connective tissue. Original magnification, ×200. Scale bar, 200 μm. (**F**) mRNA expression of fibronectin (*Fn*). mRNA expressions were normalized to *Rn18S* mRNA expression. Bars indicate means ± SDs. ** *p* < 0.01 vs. control, *** *p* < 0.001 vs. control, ^§^ *p* < 0.05 vs. diabetic ^§§^ *p* < 0.01 vs. diabetic, ^§§§^ *p* < 0.001 vs. diabetic (*n* = 6/group).

Diabetic rats had an increased LV mRNA expression of transforming growth factor beta (TGF-β, *Tgfb1*) and connective tissue growth factor (CTGF, *Ccn2*). Low-dose RAASi treatment attenuated the elevation of the investigated markers (Figure 4A–C). In parallel, the DM-induced higher fibronectin (*Fn*) expression was abolished by RAASis, indicating a milder myocardial remodeling (Figure 4F).

Picrosirius red staining was performed to evaluate the extent of LV myocardial collagen content. The analysis revealed increased intramyocardial collagen deposition in the diabetic group. The administration of RAASis significantly reduced collagen accumulation (Figure 4D,E).

## 3. Discussion

DM reduces the life expectancy of millions of people and affects the quality of life even more [19]. DM and CV disease are associated with multiplicative mortality. A person in their sixties who has both conditions has a reduction in life expectancy of 15 years. This is even more dramatic for patients at a younger age [20,21]. Improved understanding and the early diagnosis of CV complications help to identify individuals for whom more aggressive therapy is needed. 

In T1DM, CV events occur more than a decade earlier due to premature atherosclerosis. Chronic hyperglycemia and dyslipidemia result in lipid, cholesterol, and calcium accumulation within the arterial walls, leading to arterial wall thickening, atherosclerotic plaque formation, and increased vascular stiffness. One measure of aortic stiffness is PWV, which estimates the pulse transit time between the carotid and femoral arteries [22]. Several studies reported increased PWV in T1DM patients compared to healthy subjects [23,24,25]. Arterial stiffness increases with the duration of T1DM, and PWV is associated with cardiovascular events and all-cause mortality, independently of other risk factors [26].

Vascular functional abnormalities are uniform along the whole aorta; however, in DM, the abdominal aorta is where the earliest changes in vascular structure occur. Some reports in STZ-induced T2DM rats on a high-fat diet demonstrated that altered elastin lamellae and increased tissue lipid deposition are mainly confined to the abdominal region. In the same model, others showed segmental differences in functional responses to endothelin and Ang II. In light of these experimental data, it is not surprising that structural and functional dissimilarities differentially influence disease vulnerability [27], and RAASis can improve arterial stiffness and PWV in hypertensive patients [28,29,30,31,32].

The American Heart Association and the American Diabetes Association emphasize the importance of maintaining normoglycemia in managing CV risk factors in DM. For patients with blood pressure higher than 130/80 mmHg, an antihypertensive drug, mostly preferred RAASis, should be started.

However, in a clinical study investigating hypertensive patients, large arteries viscoelasticity and functions were impaired, but the alterations in elasticity differed between hypertensive individuals with or without T2DM. Furthermore, antihypertensive treatment with ramipril led to more robust viscoelastic beneficial changes in patients with T2DM despite a similar reduction in pressure values [33].

Novel biomarkers exemplified by cardiac troponin I or BNP are potent predictors of LV dysfunction and CV outcomes. BNP is primarily released in response to LV volume expansion and pressure overload [34,35]. Cardiac troponin I, a regulatory component of the contractile apparatus in myocardial cells, is accepted as a standard biomarker for diagnosing and predicting myocardial injury [36]. BNP and troponin I have been increased in diabetic patients and experimental DCM models [37,38,39]. However, there is less known about the effects of RAASis on the changes in these biomarkers. A study showed that AngII receptor type 1 agonist prevents DM-induced troponin I protein kinase C phosphorylation. It has been recently published that captopril and losartan diminish the increased level of troponin I and infarct size in STZ-diabetic rats after myocardial injury [40]. In spontaneously hypertensive rats, BNP levels were reduced in losartan-treated animals [41], and in studies of acute myocardial infarction injury, ramipril and eplerenone prevented BNP elevation [42,43].

In humans, the PONTIAC I study showed that, in T2DM patients without clinical symptoms of cardiac disease and with high BNP levels, intensified therapy with ACEi (100% of the recommended dose) prevented cardiovascular events without changing BNP levels. The ongoing PONTIAC II will clarify whether these benefits are more significant in patients with BNP elevation than those with average BNP levels (NCT02817360). Another ongoing study (ADOPT) investigated the efficacy of intensified ACEi or ARB therapy in preventing CV events in T2DM patients with high CV risk. In the MIRAD trial, the high-dose eplerenone add-on therapy to ARB/ACEi was associated with a reduction in BNP level, LV mass, and extracellular volume, suggesting an alteration in cardiac remodeling and fibrosis [44].

Cardiac fibrosis is the final common pathway of the development of heart failure. Clinical studies have demonstrated the presence of myocardial fibrosis in patients with DM, which may manifest independently of hypertension or coronary atherosclerosis [45,46]. Hyperglycemia-induced AngII and aldosterone activity promote the deposition of ECM proteins and extensive myocardial fibrosis, leading to cardiac remodeling and impaired function [47,48,49]. Regarding the antifibrotic effects of RAASis, previous preclinical studies in STZ-induced diabetic models have demonstrated the attenuations in myocardial interstitial fibrosis [50,51,52]. However, these studies used regular or high doses of RAASis [53,54,55]. Recent data from the HOMAGE trial investigating the effects of spironolactone on fibrosis and cardiac function in people at increased risk of developing heart failure showed that serum procollagen type I C-terminal propeptide is lower in spironolactone-treated patients. However, no subgroup analyses investigated whether these effects were explicitly reflected in the DM population [56]. These data further strengthen our hypothesis that RAASis might be protective independent of the blood pressure-lowering effect. In T1DM kidneys, we have previously shown that RAASis halt renal fibrosis progression [14].

Concerning the heart and vasculature, apart from a single study demonstrating that low-dose losartan normalized PWV in iron-overloaded rats [57], as far as we are aware, these are the first data in T1DM regarding the cardioprotective effect of low-dose RAASis, especially of MR antagonists in monotherapy. Here, we demonstrated that the oral administration of low-dose RAASis alters functional vascular impairment and decreases myocardial injury, including fibrosis. To test whether these cardioprotective properties are associated with, or limited to, the blood pressure-lowering effects of RAASis, we chose the treatment doses that do not affect blood pressure based on our previous studies [14,58]. We confirmed that neither DM nor RAASi affected blood pressure, providing evidence in support of the non-depressor drug doses used in the current protocol. More importantly, we found that low-dose RAASis can improve CV function by halting the progression of arterial stiffness.

BNP and troponin I robustly increased in the T1DM rats, confirming the myocardial injury and LV distress even without blood pressure changes. Treatment with various RAASis reduced the mRNA expression of *Nppb* and serum troponin I, indicating milder myocardial damage. We also demonstrated that RAASis reduced the increased intramyocardial collagen deposition in the LV of diabetic rats, but only eplerenone treatment reduced fibrotic tissue accumulation in the aortic media, parallel with the decrease in IMT.

In recent years, Klotho, produced in the kidney, has gained attention as a potentially sensitive and specific biomarker in renal disease with cardiac complications. It is part of a unique endocrine system that regulates the inactivation of oxidative stress, inflammation, and fibrotic pathways in the kidney and heart [59,60]. Klotho deficiency is a pathogenic CV disease factor associated with arterial stiffness, LV hypertrophy, and cardiac remodeling. A decline in serum Klotho levels, followed by an increase in FGF23 levels, can serve as an early biomarker for kidney dysfunction and a predictor of CV disease risk [61]. Patients with T1DM have lower Klotho levels than the normal population, related to higher IMT [62]. Moreover, the haplodeficiency of the Klotho gene increased collagen deposition in the media of the aorta, suggesting that serum levels of Klotho could be closely related to fibrotic changes in the aorta [63,64].

We showed that serum Klotho was lower, while LV FGF23 levels were higher in diabetic rats than in controls. This correlates with the few data measured in T1DM animal models and diabetic patients [65,66]. All RAASis decreased FGF23 protein levels, while only eplerenone reversed the decline in serum levels of Klotho and reduced fibrosis in the aorta. This aligns with the literature, where Klotho-deficiency-related changes were blocked via the eplerenone treatment [64]. The mechanism may be the same as that recently shown in renal fibrosis. High aldosterone levels downregulate Klotho transcriptional activity by increasing histone deacetylase expression. This, in turn, leads to the deacetylation of histone H3K9, a site associated with the Klotho gene promoter, suggesting that various RAASis differentially affect the Klotho/FGF23 axis; this may explain our results showing the sole efficacy of eplerenone on aortic fibrosis [67]. These results may encourage the importance of the monotherapeutic use of eplerenone and other MR antagonists in preventing cardiac fibrosis. It is noteworthy to mention that, in a recent study involving patients at risk for HF with CKD and T2DM, irrespective of their HF history, the selective MR antagonist finerenone, when added to the maximally tolerated dose of RAASis, demonstrated improvement in CV and kidney outcomes [68].

In conclusion, our study demonstrates that non-depressor doses of RAASis effectively mitigate hyperglycemia-induced cardiovascular complications without impacting blood pressure levels. RAASis in monotherapy improve vascular function and halt cardiac tissue remodeling in STZ-induced CV changes. The comparative analysis of various RAASis suggests that monotherapy with MR antagonist eplerenone can offer equal or even superior efficacy compared to ACEis or ARBs in arresting the progression of arterial and myocardial injuries in T1DM.

We identified a possible novel target, Klotho, with which eplerenone decreases fibrotic tissue accumulation in the media of the aorta. Ultimately, a better understanding of these effects has the potential to pave the way for novel therapeutic approaches and benefit clinical practice. However, further human clinical trials conducted under controlled conditions are essential to validate these suggestions and assess the risk–benefit profile, particularly in the case of MR antagonists.

## 4. Materials and Methods

### 4.1. Ethical Approval

Animal procedures were conducted according to the regulations of the Committee on the Care and Use of Laboratory Animals of the Council on Animal Care at Semmelweis University, Budapest, Hungary (PEI/001/380-4/2013).

### 4.2. Materials

Chemical substances and reagents were procured from Sigma-Aldrich (Budapest, Hungary), and standard plastic laboratory materials were obtained from Sarstedt (Nümbrecht, Germany) unless otherwise specified.

### 4.3. Experimental Design

Eight-week-old male Wistar rats (Rattus norvegicus), weighing 200 ± 10 g, were obtained from the “Toxi-Coop” Toxicological Research Center (Dunakeszi, Hungary). The rats were housed in groups of three in plastic cages under a 12 h light/dark cycle at a constant temperature of 24 ± 2 °C and humidity of 55%. They were given ad libitum access to standard rodent chow and tap water. The rats underwent a one-week acclimatization period before the start of the experiment. A qualified individual monitored the health and well-being of the animals daily.

T1DM was induced with a single intraperitoneal injection of 65 mg/bwkg streptozotocin (STZ) dissolved in 0.1 M citrate buffer (pH 4.5). After overnight fasting, blood glucose levels were measured three times from the tail vein using a Dcont IDEAL device (77 Elektronika, Budapest, Hungary). Rats with a peripheral blood glucose value above 15 mmol/L 72 h after the STZ injection were enrolled in the study. Five weeks after the onset of T1DM, the rats were randomized into four groups (*n* = 6 animals/group). They were orally treated for two weeks with the following substances: isotonic saline as vehicle (D) or angiotensin-converting enzyme inhibitor (ACEi) ramipril (D + RAM, 10 µg/bwkg/day) or AngII type 1 receptor blocker (ARB) losartan (D + LOS, 20 mg/bwkg/day) or mineralocorticoid receptor (MR) antagonist eplerenone (D + EPL, 50 mg/bwkg/day). The dosages of RAASis were based on previous experiments, ensuring the effective blockade of RAAS without affecting systemic blood pressure [14,58]. As controls, non-diabetic, as well as age- and body-weight-matched healthy animals (Control; *n* = 6 animals/group), received an equivalent volume of citrate buffer without STZ once and the same amount of saline through oral gavage daily. They followed the same treatment duration as the diabetic animals.

At the end of the experimental protocol, the rats were anesthetized using a combination of 75 mg/bwkg Ketamine (Richter Gedeon, Budapest, Hungary) and 10 mg/bwkg Xylazine (Medicus Partner, Biatorbagy, Hungary) and then sacrificed by drawing terminal blood. Blood, urine, heart, and aorta samples were collected, and left ventricular (LV) tissues were dissected. All samples were promptly snap-frozen or placed in formalin for further investigation.

### 4.4. Measurement of Arterial Blood Pressure and PWV

**Figure 5:**
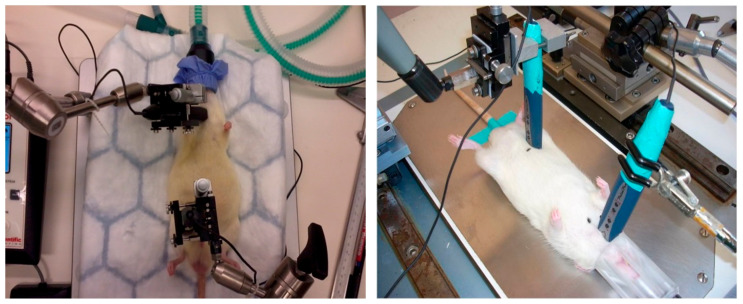
Non-invasive pulse wave velocity measurement with PulsePenLab applanation tonometry device in anesthetized Wistar rat.

After two weeks of treatment, PWV and blood pressure were measured under isoflurane (1.5% maintenance after 4% induction concentration, Ghislandi es Tarsai Kft., Budapest, Hungary) anesthesia on a 37 °C heating pad. Systolic and diastolic blood pressure were non-invasively measured on a tail vein, and the mean arterial pressure (MAP) was calculated using the CODA tail cuff standard monitor system (EMKA Technologies, Paris, France), which utilizes clinically validated volume pressure recording technology. Non-invasive PWV registration was performed using PulsePenLab applanation tonometry device (DiaTecne, Milan, Italy), simultaneously recording the electrocardiogram. The pulse wave was detected over the carotid and femoral arteries on the same side. A high-precision digital caliper was used to measure the direct distance from the carotid point and the peripheral point of application of the tonometric probes. The time difference between the two waves was divided by 80% of the direct carotid–femoral surface distance to calculate the PWV (Figure 5) [69].

### 4.5. Metabolic Parameters

Body and heart weights were measured, and the heart-to-body weight ratios were calculated. Serum glucose and total cholesterol were determined photometrically with commercially available kits on a Hitachi 912 chemistry analyzer (Roche Hitachi, Basel, Switzerland).

### 4.6. ELISA

Blood samples were collected and centrifuged at 3600 rpm for 6 min. The serum samples were diluted to a 1:10 ratio. Commercially available rat-specific sandwich ELISA kits (Abcam, Cambridge, UK, and ABclonal, Woburn, MA, USA) were used to measure serum cardiac troponin I and Klotho levels, following the manufacturer’s protocols. The concentration was determined by measuring the absorbance at 450 nm with wavelength correction at 650 nm using a SPECTROstar Nano microplate reader (BMG Labtech, Ortenberg, Germany).

### 4.7. Histology

Elastic fibers in the aorta were investigated using Orcein staining. Aorta sections were dissected, fixed in 8% paraformaldehyde, and embedded in paraffin for immunohistochemistry. Histological examination was conducted under 20× objective magnification using Case Viewer 2.4 (3DHISTECH, Budapest, Hungary). The aorta’s IMT was measured on cross-sections, and the mean value of ten measurements was calculated.

To determine collagen content and perivascular fibrosis in the left ventricle, picrosirius red staining was used. Fresh-frozen heart tissues were sectioned with 4 μm thickness and stained, and the slides were digitalized with a Pannoramic1000 slide scanner (3DHistech, Budapest, Hungary). The intramyocardial collagen was measured with the Quant Center HistoQuant 2.5 module (3DHistech, Budapest, Hungary) using specific RGB pixel intensity parameters. The measurements were performed manually cautiously to avoid the endocardium and perivascular connective tissue. The surface of measurement was 1.6 mm^2^ in each heart.

Masson’s trichrome staining was performed on 5 μm thick aortic sections to evaluate the medial fibrotic tissue accumulation. The specifically stained interstitial areas were measured within the intima–media layer on each aortic cross-section. The number of pixels containing blue-stained fibrotic tissue was divided by the number of other pixels in the area to obtain the ratio of medial fibrosis using ImageJ (Version 1.37; US National Institute of Health, Bethesda, MD, USA).

### 4.8. Lyophilization and Product Processing

Heart tissue samples were collected and stored at −80 °C. For lyophilization, samples were optimally arranged to maximize surface exposure and freeze-dried using a ScanVac CoolSafe Touch Superior device (LaboGene A/S, Allerod, Denmark). Tubes were left open throughout the whole process. Pre-freezing was carried out at −40 °C for 1 h, followed by primary drying in six 2 h steps at 0.22 hPa with a gradual increase in temperature up to 30 °C. Secondary drying was performed at 0.1 hPa and 40 °C for 3 h. The resulting dried tissue products were manually smashed with 20 Gauge needles and further pulverized using a TissueLyser LT (Qiagen GmbH, Hilden, Germany). The powdered tissue samples were stored at 4 °C until the measurements.

### 4.9. Western Blot

All reagents were obtained from Bio-Rad Laboratories (Hercules, CA, USA) for Western blot analysis. Total protein was extracted from lyophilized left ventricular muscle homogenates using a lysis buffer (1 M Tris, 0.5 M EGTA, 1% Triton X-100, 0.25 M NaF, 0.5 M phenylmethylsulfonyl fluoride, 0.5 M sodium orthovanadate, 5 mg mL^−1^ leupeptin, and 1.7 mg mL^−1^ aprotinin, pH 7.4). The protein concentrations were measured with a detergent-compatible Bradford dye-binding method protein assay kit. Samples containing 30 μg protein/lane were loaded onto 4–20% gradient Mini-PROTEAN TGX polyacrylamide precast gels, separated via electrophoresis, and transferred to nitrocellulose membranes. Membranes were then blocked in 5% *w*/*v* non-fat dried milk in Tris-buffered saline (TBS) for 1 h at room temperature and immunoblotted with specific primary antibodies against FGF23 (AB56326, Abcam, Cambridge, UK) at 4 °C overnight. After washing, the membranes were incubated with the appropriate HRP-conjugated secondary antibodies. Chemiluminescence was detected using a LuminataTM Forte (Millipore Corporation, Billerica, MA, USA) substrate, and immunoreactive bands were quantified densitometrically on Versadoc, Quantity One 1-D Analysis software (Version 4.6.8; Bio-Rad, Budapest, Hungary) as integrated optical density (IOD) after background subtraction. The IOD was factored for Ponceau S staining to ensure equal protein loading. The protein abundance is represented as IOD/Ponceau S/internal control.

### 4.10. Quantitative Reverse Transcription Polymerase Chain Reaction (RT-qPCR)

Total RNA was extracted from rat LV tissue using a Total RNA Isolation Mini Kit (Geneaid Biotech, New Taipei City, Taiwan). The quality and quantity of isolated RNA were measured on a NanoDrop ND-1000 spectrophotometer (Baylor College of Medicine, Huston, TX, USA). For cDNA synthesis, 250 ng of RNA was reverse-transcribed using a First-Strand cDNA Synthesis Kit for RT-PCR (Thermo Fisher Scientific, Waltham, MA, USA). qPCR was then performed using SYBR Green I Master enzyme mix (Roche Diagnostics, Indianapolis, IN, USA) and specific primers (Invitrogen, Budapest, Hungary) based on sequences from the National Center for Biotechnology Information’s nucleotide database (Table 2). Data analysis was conducted using LightCycler 480 software version 1.5.0 (Roche Diagnostics). mRNA expressions of *Nppb*, *Tgfb1*, *Ccn2*, *Pdgfb*, and *Fn* were normalized to the mRNA expression of the ribosomal 18S (Rn18s) housekeeping gene from the same samples, serving as the reference transcript.

### 4.11. Statistical Analysis

Data are presented as mean ± standard deviations (SD). Statistical analysis was conducted using Prism software (version 10.1.0; GraphPad Software, San Diego, CA, USA). Multiple comparisons and interactions were assessed using one-way ANOVA followed by the Holm–Sidak post hoc test. The Kruskal–Wallis ANOVA on ranks, followed by Dunn’s correction, was applied for non-parametric data. A significance level of *p* < 0.05 was considered statistically significant.

## 5. Conclusions

We found that low-dose RAASis effectively improve vascular function and halt cardiac tissue remodeling in T1DM without affecting blood pressure. Monotherapy with MR antagonist eplerenone can offer equal or superior efficacy compared to ACEis or ARBs in halting the progression of arterial and myocardial injuries. By elevating Klotho levels, we identified a novel mechanism of action for eplerenone, with which it decreases IMT and fibrotic tissue accumulation in the aorta.

## 6. Study Limitations

A limitation of our study was the lack of mechanistic investigations using in vitro systems. Future studies integrating mechanistic analyses may provide valuable insights that complement our findings and provide a better understanding of the underlying molecular mechanisms.

## Figures and Tables

**Table 1:** Non-fasting serum glucose, body weight, mean arterial pressure, heart rate, heart weight, and heart-to-body weight ratio of control, diabetic (D), ramipril (D + RAM)-treated, losartan (D + LOS)-treated, or eplerenone (D + EPL)-treated diabetic rats. All data are represented as means ± SDs. * *p* < 0.05 vs. control, *** *p* < 0.001 vs. control (*n* = 6/group).

	Control	Diabetic (D)	D + RAM	D + LOS	D + EPL
Non-fasting serum glucose (mmol/L)	12.8 ± 1.68	37.5 ± 7.36 ***	40.9 ± 3.36 ***	41.9 ± 6.45 ***	33.2 ± 2.45 ***
Body weight (g)	327 ± 19.7	262 ± 35.4 ***	258 ± 13.3	247 ± 29.4	257 ± 38.8
Mean arterial pressure (mmHg)	77.4 ± 11.9	76.2 ± 6.86	76.5 ± 11.8	74.5 ± 17.2	77.7 ± 17.1
Heart rate (bpm)	479 ± 58.6	371 ± 23.9 ***	375 ± 22.9 ***	375 ± 12.9 ***	329 ± 22.0 ***
Heart weight (g)	1.10 ± 0.06	1.05 ± 0.14	1.1 ± 0.13	0.97 ± 0.10	1.05 ± 0.10
Heart-to-body weight ratio (%)	0.34 ± 0.03	0.40 ± 0.02 *	0.43 ± 0.04 *	0.39 ± 0.05	0.41 ± 0.05 *

**Table 2:** The primer pairs are used for quantitative RT-qPCR.

Gene	NCBI ID	Primer Pairs		Product Length
*Nppb*	NM_031545.1	Forward:	5′-CAG CTC TCA AAG GAC CAA GG 3′	192 bp
		Reverse:	5′-CTA AAA CAA CCT CAG CCC GT 3′	
*Tgfb1*	NM_021578.2	Forward:	5′-GCACCGGAGAGCCCTGGATACC 3′	222 bp
		Reverse:	5′-CCCGGGTTGTGTTGGTTGTAGAGG 3′	
*Pdgfb*	NM_031524.1	Forward:	5′-TCGATCGCACCAATGCCAACTTCC 3′	236 bp
		Reverse:	5′-CACGGGCCGAGGGGTCACTACTGT 3′	
*Ccn2*	NM_022266.2	Forward:	5′-TCCACCCGGGTTACCAATGACAATAC 3′	195 bp
		Reverse:	5′-CTTAGCCCGGTAGGTCTTCACACTGG 3′	
*Fn*	NM_019143.2	Forward:	5′-GGATCCCCTCCCAGAGAAGT 3′	188 bp
		Reverse:	5′-GGGTGTGGAAGGGTAACCAG 3′	
*Rn18s*	NR_046237.1	Forward:	5′-GCG GTC GGC GTC CCC CAA CTT CTT-3′	105 bp
		Reverse:	5′-GCG CGT GCA GCC CCG GAC ATC TA-3′	

## Data Availability

Data are contained within the article.
